# Self-Renewing *Pten^-/-^TP53^-/-^* Protospheres Produce Metastatic Adenocarcinoma Cell Lines with Multipotent Progenitor Activity

**DOI:** 10.1371/journal.pone.0026112

**Published:** 2011-10-11

**Authors:** Wassim Abou-Kheir, Paul G. Hynes, Philip Martin, Juan Juan Yin, Yen-Nien Liu, Victoria Seng, Ross Lake, Joshua Spurrier, Kathleen Kelly

**Affiliations:** 1 Cell and Cancer Biology Branch, National Cancer Institute, National Institutes of Health, Bethesda, Maryland, United States of America; 2 Department of Anatomy, Cell Biology and Physiological Sciences, Faculty of Medicine, American University of Beirut, Beirut, Lebanon; Florida International University, United States of America

## Abstract

Prostate cancers of luminal adenocarcinoma histology display a range of clinical behaviors. Although most prostate cancers are slow-growing and indolent, a proportion is aggressive, developing metastasis and resistance to androgen deprivation treatment. One hypothesis is that a portion of aggressive cancers initiate from stem-like, androgen-independent tumor-propagating cells. Here we demonstrate the in vitro creation of a mouse cell line, selected for growth as self-renewing stem/progenitor cells, which manifests many in vivo properties of aggressive prostate cancer. Normal mouse prostate epithelium containing floxed Pten and TP53 alleles was subjected to CRE-mediated deletion in vitro followed by serial propagation as protospheres. A polyclonal cell line was established from dissociated protospheres and subsequently a clonal daughter line was derived. Both lines demonstrate a mature luminal phenotype in vitro. The established lines contain a stable minor population of progenitor cells with protosphere-forming ability and multi-lineage differentiation capacity. Both lines formed orthotopic adenocarcinoma tumors with metastatic potential to lung. Intracardiac inoculation resulted in brain and lung metastasis, while intra-tibial injection induced osteoblastic bone formation, recapitulating the bone metastatic phenotype of human prostate cancer. The cells showed androgen receptor dependent growth in vitro. Importantly, in vivo, the deprivation of androgens from established orthotopic tumors resulted in tumor regression and eventually castration-resistant growth. These data suggest that transformed prostate progenitor cells preferentially differentiate toward luminal cells and recapitulate many characteristics of the human disease.

## Introduction

Prostate cancers (PC) display a range of clinical behavior; most are relatively slow-growing tumors of minor clinical significance, while about 10% develop to aggressive disease with poor prognosis [1]. Human prostate adenocarcinoma has a mature luminal phenotype distinguished by androgen receptor (AR) expression and androgen-dependent survival. Progressive PC is characterized by the ability to grow in androgen deprived conditions and to metastasize, primarily to the bone, resulting in osteoblastic lesions [2]. Understanding the biology of aggressive disease and the cellular origins of castrate-resistant PC is a major goal of PC research.

The cancer stem cell (CSC) model posits a hierarchical organization of tumors, with self-renewing stem-like cells that generate progeny, which subsequently undergo epigenetically programmed differentiation and loss of tumorigenicity [3], [4], [5]. The applicability of the CSC model for human prostate cancer is not known. However, the presence of stem-like cells in human prostate cancer xenografts and cell lines as well as in mouse models has suggested that prostate cancer may contain epithelial populations with different extents of lineage commitment [6], [7], [8], [9], [10].

Another consideration with respect to prostate cancer phenotypes is the potential cancer cells-of-origin. As has been shown for breast cancer, the differentiation stage of the progenitor cell of origin significantly influences the histological subtype of tumors and their clinical behavior [11], [12], [13]. It has been implied, by two different studies, that both basal and luminal progenitors can give rise to adenocarcinoma and therefore act as the prospective cell of origin for PC [14], [15], although the lineage relationship of the two target progenitor cells is not entirely clear. The lack of specific cell-surface lineage markers for prostate epithelium has limited the ability to define the number and relationship of prostate progenitors and to obtain their significant enrichment.

The majority of prostate cancers have the appearance of transformed luminal cells. We hypothesize that the prostate cancer cell of origin will affect the lineage composition of minor tumor cell populations and consequently the selective mechanisms contributing to the aggressive phenotype and the acquisition of castrate-resistance. Although multiple mechanisms appear to contribute to castrate-resistant prostate cancer (CRPC), it has been hypothesized that one origin might be immature, AR negative, tumor-propagating cells, similar to multipotent progenitors [16], [17], [18]. The transformation of cells closer to the stem cell apex in lineage hierarchy might be expected to increase the likelihood of immature progenitor cells in tumors.

Underlying mutations are known to be major determinants in the clinical behavior of tumors. Loss of PTEN and aberrations of TP53 are implicated in aggressive forms of human PC. One of the most common alterations in prostate cancer, which occurs in >70% of human prostate cancers, is loss of expression of the PTEN tumor suppressor, and biallelic deletion of PTEN is correlated with CRPC [19]. Although alterations to TP53 previously have been thought to be exclusively associated with advanced disease, a recent genomic profiling study of mostly primary prostate cancers demonstrated that 24% of cases had either a hetero- or homozygous copy number loss of TP53 [20]. Consistent with abnormal TP53 contributing to PC progression, other large scale studies using combined immunohistochemistry and sequencing approaches have shown that TP53 mutations occur in approximately 5% of primary tumors and at much higher frequencies in lymph node metastases (16%) and castrate-resistant (26%) tumors [21], [22]. Additionally, TP53 mutations were found to be independent predictors of tumor recurrence in low and intermediate grade cancers.

Modeling prostate cancer in the mouse has shown that prostate epithelial cell-specific loss of Pten and TP53 results in aggressive, lethal disease that is significantly more penetrant and rapidly developing than prostate cancer resulting from Pten deletion alone [23], [24]. In vitro assays of progenitor self-renewal as measured by serial propagation of protospheres demonstrated greatly increased self-renewal activity in Pten/TP53 null relative to wild type prostate epithelium [25]. Adenocarcinoma is the main initial tumor type that arises in the prostate epithelial Pten/TP53 null model. Furthermore, adenocarcinoma tumors were observed most often upon orthotopic transplantation of Pten/TP53 null protospheres [25].

In order to originate a model of progenitor-driven prostate cancer, we initiated deletion of tumor suppressor genes in vitro in a functionally-defined progenitor population, namely protospheres. Following additional serial protosphere propagations, cell cultures were established. Here we characterize a cell line and its clonal derivative arising from such an approach. Both cell lines display AR^+^ luminal phenotypes while maintaining evidence of a progenitor population. Of interest, without prior in vivo selection, these lines express many properties of aggressive prostate cancer including metastatic ability, the generation of an osteoblastic response upon growth in bone, and the potential for castrate-resistant growth.

## Results

### Isolation and characterization of prostatic epithelial cell lines from the *Pten/TP53* conditional knock-out model

**Figure 1:**
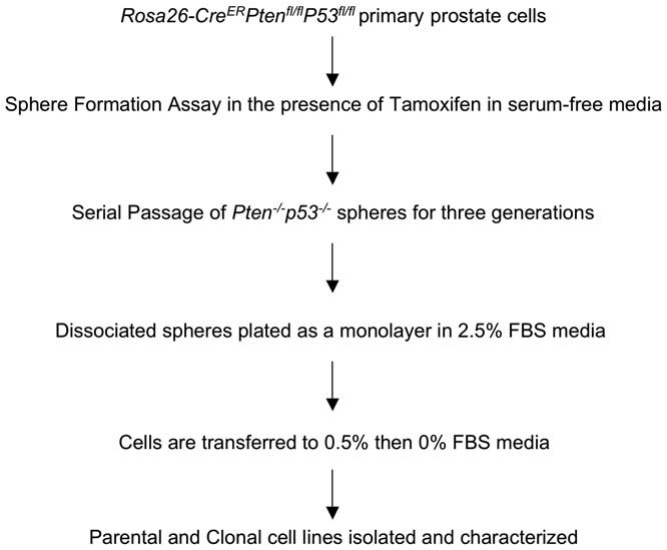
Isolation of *Pten^-/-^TP53^-/-^* cell lines from *Pten-/-TP53-/-* prostate spheres. An illustration of the generation of prostate epithelial cell lines. Cells were subcloned and the parental cells are referred to as PLum-P (Prostate Luminal-Parental) and the clone as PLum-C (Prostate Luminal-Clone).

To determine the characteristics of prostate epithelial progenitor cell lines with genomic loss of *Pten* and *TP53* we made use of a tamoxifen inducible Cre murine model containing floxed *Pten* and *TP53* alleles (Fig. 1). Single cell suspensions of primary prostatic cells isolated from intact *Rosa26-Cre^ER^Pten^fl/fl^P53^fl/fl^* mice were suspended in Matrigel™ together with serum-free minimal media and tamoxifen. The protospheres that formed were serially passaged for three generations to further enrich for stem/progenitor cells with self-renewal activity. Subsequently, the protospheres were dissociated and cells were cultured as monolayers in the presence of 2.5% FBS. After establishing a monolayer culture, cells were weaned off serum until they grew in serum-free media, where no exogenous androgen was added to the growth media. The polyclonal parental cells, PLum-P, were used to generate daughter clones derived from single cells. The clonal line, PLum-C, was selected for further analysis based upon demonstrating the ability to form protospheres.

**Figure 2:**
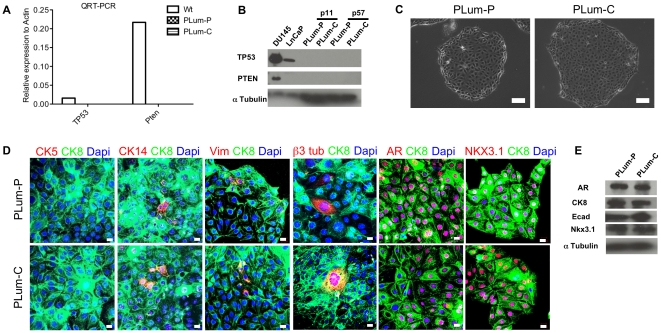
Lineage characterizations of *Pten^-/-^TP53^-/-^* cell lines. (**A**) *Pten* and *TP53* expression was determined using QRT-PCR analysis and values were normalized to *Actin*. (**B**) PTEN and TP53 expression was determined using WB analysis. (**C**) Representative bright field images of PLum-P and PLum-C cells. Scale bar  = 100 µm. (**D**) Representative immunofluorescent images of PLum-P and PLum-C cells stained for the indicated antibodies and Dapi are shown. Scale bars  = 20 µm. (**E**) WB analysis for the indicated proteins in Plum-P and Plum-C cells is shown.

A quantitative reverse transcription-PCR (QRT-PCR) and Western Blot (WB) analyses were performed to characterize the genotype of the cells. QRT-PCR and WB data confirmed the deletion of *Pten* and *TP53* in both PLum-P and PLum-C cell lines (Fig. 2A and 2B). To determine the cellular phenotype of these established cell lines, microscopic analyses using markers for the major cell types in the prostate epithelium were performed. PLum-P and PLum-C cells showed typical epithelial cell morphology with a cobble-stone appearance and well-defined cell boundaries (Fig. 2C). PLum-P and PLum-C cells were stained for lineage markers including CK5 and CK14, CK8, and β3 tubulin, which are characteristically expressed in basal, luminal and neuroendocrine cells, respectively. While all the cells expressed CK8, CK5 was not detected and minor populations of cells co-expressed CK14 and CK8 (less than 5%) and Vimentin and CK8 (less than 1%) (Fig. 2D). Furthermore, very rare neuroendocrine (β3 tubulin +ve) cells were detected. In addition to CK8, both cell lines expressed heterogenous levels of AR and the prostate luminal epithelial marker NKX3.1, suggesting the existence of mature and immature CK8^+^ cells (Fig. 2D). Moreover, WB analysis showed the expression of AR, CK8, NKX3.1 and E-cadherin in both Plum-P and Plum-C cells (Fig. 2E). Given the clonal nature of the PLum-C line, these data show that a predominantly luminal epithelial phenotyped cell line has retained capacity to produce cells expressing basal and neuroendocrine markers, and imply that immature cells with differentiative ability exist within the culture.

### PLum-P and PLum-C sphere-forming cells retain self-renewal ability and differentiation plasticity in vitro

**Figure 3:**
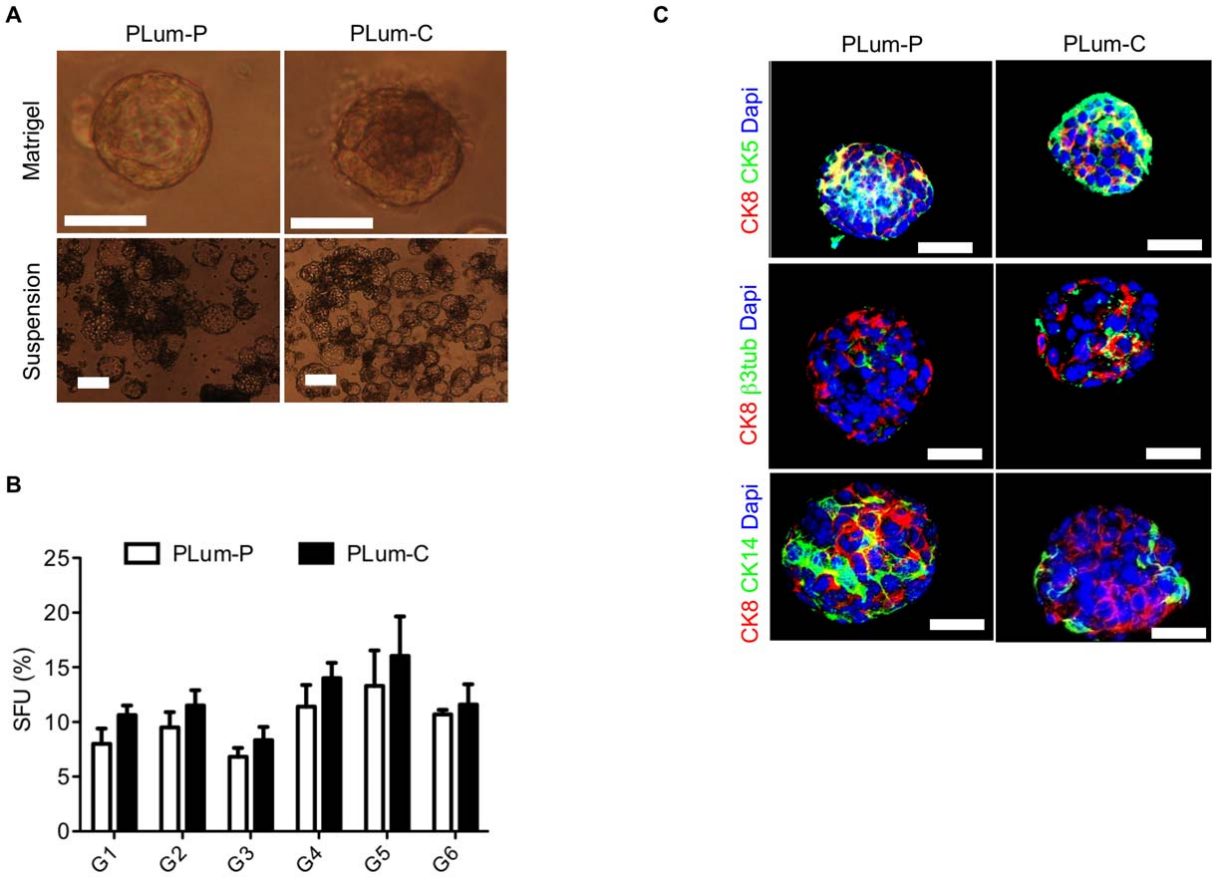
*Pten^-/-^TP53^-/-^* cell lines displaying stem-like cell properties. (**A**) Representative bright field images of PLum-P and PLum-C protospheres in Matrigel™ (upper panel) and in suspension (lower panel) are shown. Scale bar  = 100 µm. (**B**) PLum-P and PLum-C cells were plated in Matrigel™ at a density of 2,000 cells/well for sphere formation assay. Sphere forming units expressed as % of 2,000 input cells at each generation obtained from serially passaged protospheres are shown. The data are reported as mean ± SD. (**C**) immunofluorescent images of confocal cross sections from representative PLum-P and PLum-C protospheres stained for CK5, CK8, CK14, β3 tubulin and Dapi. Scale bar  = 50 µm.

To determine whether the cell lines have stem/progenitor cell-like properties, including self-renewal ability and differentiation potential, we used a sphere formation assay previously shown to select for the growth of prostate epithelial stem/progenitor cells in vitro. In addition to forming protospheres in an anchorage-independent manner (absence of Matrigel™), both cell lines formed protospheres at comparable efficiency (8% for PLum-P and 10.6% for PLum-C) when suspended in serum free media in the presence of a semi-solid basement membrane-Matrigel™, suggesting the existence of cell populations expressing stem/progenitor characteristics (Fig. 3A and 3B). Furthermore, both PLum-P and PLum-C cells serially propagated as protospheres without losing sphere forming capacity, indicating stable self-renewal ability (Fig. 3B). To assess differentiation potential, PLum-P and PLum-C protospheres were stained for lineage markers including CK5, CK8, CK14, and β3 tubulin. Confocal images through PLum-P and PLum-C protospheres costained for CK5/CK8, CK14/CK8 or β3 tubulin/CK8 are shown in Figure 3C. Interestingly, while no CK5 was detected in monolayer cultures, approximately 20–25% of PLum-P and PLum-C protospheres contained cells demonstrating CK5, CK8, and CK5^+^/CK8^+^ expression. Furthermore, almost 75% of PLum-P and PLum-C protospheres contained cells expressing CK14, CK8, and CK14^+^/CK8^+^. Moreover, β3 tubulin positive cells were detected in occasional protospheres. These data suggest that the microenvironment provided by Matrigel™ induces differentiation toward the basal lineage that is rarely observed in monolayer culture and demonstrates that protosphere-forming cells have maintained multipotent differentiation plasticity.

### PLum-P and PLum-C cells form adenocarcinoma in vivo containing the three prostate epithelial cell lineages

**Figure 4:**
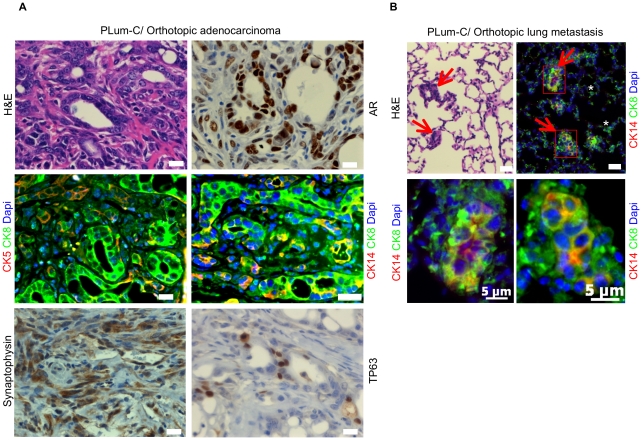
Orthotopic injection of PLum-C cells results in adenocarcinoma and lung metastases displaying variable degrees of luminal epithelial differentiation. (A) Orthotopic tumors were generated by injecting PLum-C cells in surgically castrated nude male mice with implanted testosterone pellet. Serial sections of orthotopic tumors were stained with H&E, AR, CK5, CK14, CK8, Synaptophysin, TP63, and Dapi. Scale bar  = 10 µm. (B) Representative sections of lung metastasis from orthotopic injection of PLum-C cells stained with H&E, CK8, CK14, and Dapi. Scale bar  = 10 µm, unless otherwise stated. Sections for immunofluorescent staining were cut at least 50 µm deeper relative to the first H&E section. Red arrowheads indicate lung metastasis, and white asterix indicate lung epithelium.

We subsequently examined the tumorigenic potential of PLum-P and PLum-C cells. Both PLum-P and PLum-C rapidly formed tumors upon subcutaneous transplantation in male nu/nu mice and the major histological type of tumor detected was adenocarcinoma with areas of poorly differentiated or sarcomatoid carcinoma (Fig. S1). The tumor phenotype was further characterized following orthotopic growth in male nu/nu mice receiving androgen supplements. The majority of tumor area formed by PLum-C orthotopic transplantation was comprised of adenocarcinoma with minor compartments of poorly differentiated or sarcomatoid carcinoma (Fig. 4A and Table 1). Immunohistological examination of serial orthotopic tumor sections was performed. PLum-C orthotopic adenocarcinoma was comprised of cells with a luminal (CK8^+^/CK14^-^/CK5^-^) and/or intermediate (CK8^+^/CK14^+^/CK5^+/-^) phenotype (Fig. 4A and Table 1). Moreover, rare cells with basal phenotype (TP63 or CK5 or CK14 only) were also detected. Orthotopic adenocarcinoma uniformly displayed strong nuclear labeling of AR. Cells expressing the neuroendocrine lineage marker synaptophysin were also present throughout the tumors and were positive for p-AKT indicating their tumorigenic origin (Fig. S2). Overall, PLum-C orthotopic adenocarcinoma tumors displayed a glandular luminal phenotype with relatively frequent occurrences of CK8/Synaptophysin double positive cells and rare occurrences of basal cells. Similarly to the various lineage markers observed in vitro, the tumor data suggest that PLum-P and PLum-C cell lines demonstrate differentiation plasticity.

**Table 1:** Summary of immunohistochemical marker expression in PLum-C orthotopically transplanted tumors with and without androgen.

	Androgen replete		Androgen deplete	
IHC Marker	Adeno (Major)	Sarc (Minor)	Adeno (Minor)	Sarc (Major)
**CK8**	+++	++↓	+++	++↓
**CK5**	++	-	++	-
**CK8+/CK5+**	++	-	++	-
**CK14**	++	++	++	+
**CK8+/CK14+**	++	++	++	+
**TP63**	+	-	+	-
**AR**	++	++↓	+/-↓	+/-↓
**Synaptophysin**	++	++	++	++
**Vimentin**	+/-	+++	+/-	+++
**Vimentin/CK8+**	+/-	++	+/-	++
**Lung Metastases**	3/8		3/6	

Adenocarcinoma (Adeno.), sarcomatoid carcinoma (Sarc.). +++ marker present in most or all cells (80%–100%), ++ marker present in many cells (50%–80%), however there are significant numbers of cells that lack expression, + marker present in a minority of cells (5%–50%), +/- marker is present in only rare populations of cells (less than or equal to 5%), - absence of marker. ↑Denotes increased and ↓denotes decreased IHC labeling intensity relative to epithelial cells in adjacent normal prostate. All lung metastasis were CK8^+^ with variable levels of CK14 expression. CK5 and Vimentin expression was rarely detected, and TP63 and AR expression was not detected.

### PLum-P and PLum-C cells have metastatic potential and induce osteoblastic bone tumor formation

**Figure 5:**
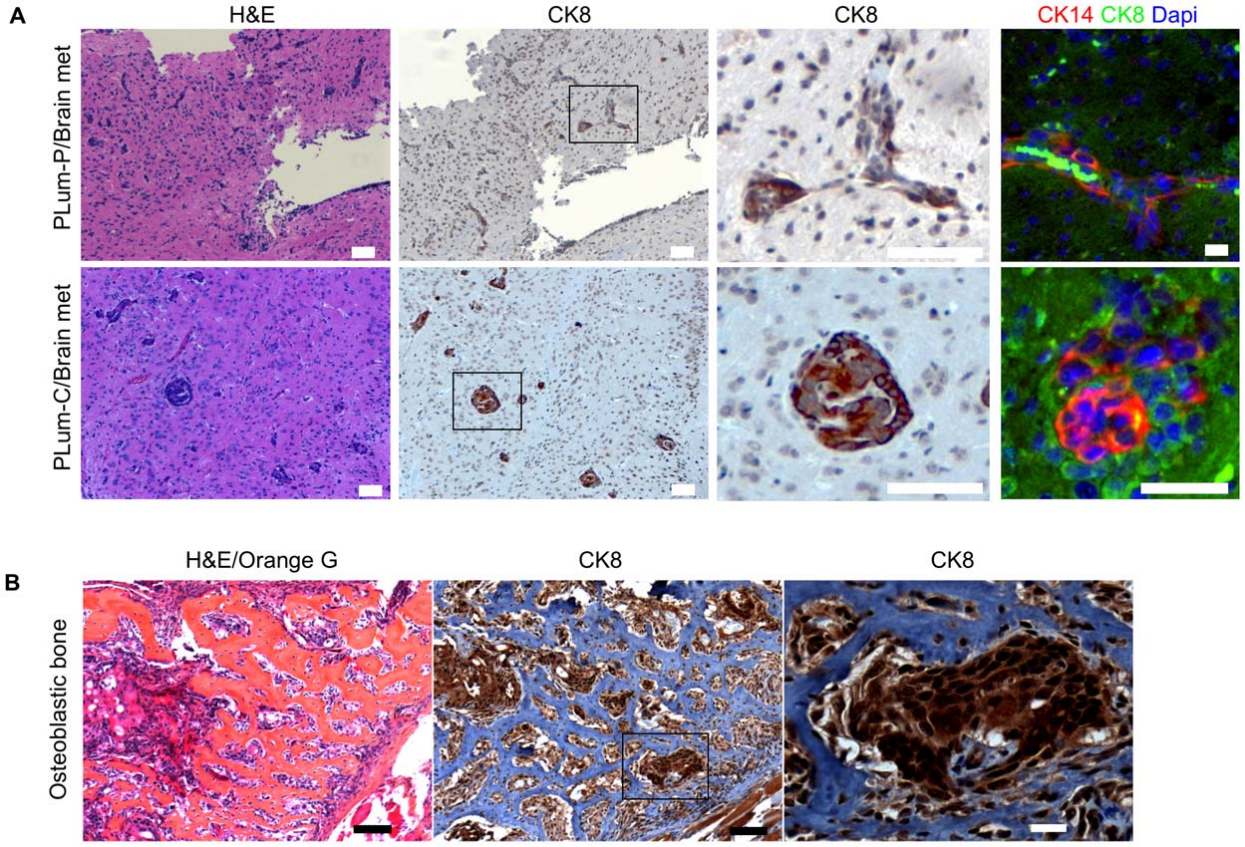
*Pten^-/-^TP53^-/-^* cells metastasize to brain following intracardiac injection and induce osteoblastic bone tumor formation upon intratibial injection. (A) Brain metastases were generated by intracardiac injection of PLum-P and PLum-C cells into nude male mice. Cross sections of brain tissues stained with H&E and labeled with antibodies against CK8, CK14, and Dapi showing metastases. Scale bar  = 10 µm. Sections for immunofluorescent staining were cut at least 50 µm deeper relative to the first H&E section. (B) Osteoblastic lesions were detected upon intratibial injection of PLum-C cells into nude male mice. Cross sections of osteoblastic tibia bones stained with H&E/Orange G and CK8. Scale bar  = 100 µm. A magnified tumor focus as indicated by a boxed area in the middle panel is shown on the right. Scale bar  = 20 µm.

The metastatic potential of the cells was investigated next. Despite no prior in vivo selection for tumorigenic growth, small undifferentiated lung metastases were observed in 3/8 mice with PLum-C orthotopic adenocarcinoma (Fig. 4B). These micrometastases were comprised of luminal (CK8) cells with varying percentages of intermediate cells (CK8^+^/CK14^+^). While synaptophysin, CK5, and Vimentin expression were present but rarely detected, TP63 and nuclear AR were not observed. These data suggest that the cells with the greatest metastatic capacity are immature luminal-lineage cells. In addition to orthotopic transplantation, an experimental metastasis assay was performed using intracardiac left ventricle inoculation. PLum-P and PLum-C cells carrying a luciferase reporter were used in order to monitor the growth over time using bioluminescent imaging. Brain metastases (12.5% frequency) were detected after three months in mice inoculated with PLum-P and PLum-C cells (Fig. 5A). Lung metastases were also detected at the same frequency. Immunohistological analysis of brain serial sections showed that the metastases were positive for CK8 and CK8^+^/CK14^+^ staining, indicating their epithelial origin. As in the case with lung metastasis from orthotopic tumors, brain metastases showed rare CK5 but no TP63 or nuclear AR expression.

A major clinical challenge in prostate cancer comes from the propensity of the progressive disease to metastasize to bone, leading to significant morbidity and eventually death. Prostate cancer bone metastasis displays characteristic osteoblastic, rather than osteolytic, lesions. To determine whether the cells produce tumors in the bone microenvironment, PLum-C cells were injected intratibially into male nu/nu mice. Using x-ray imaging, osteoblastic lesions were detected, as early as eight weeks post injection, in 50% of inoculated mice. Histological analysis was used to confirm the presence of osteoblastic lesions induced by PLum-C cells, and serial sections were stained for CK8 to show the epithelial origin of the cells (Fig. 5B).

### In vitro androgen dependence and the development of castrate resistance in vivo

**Figure 6:**
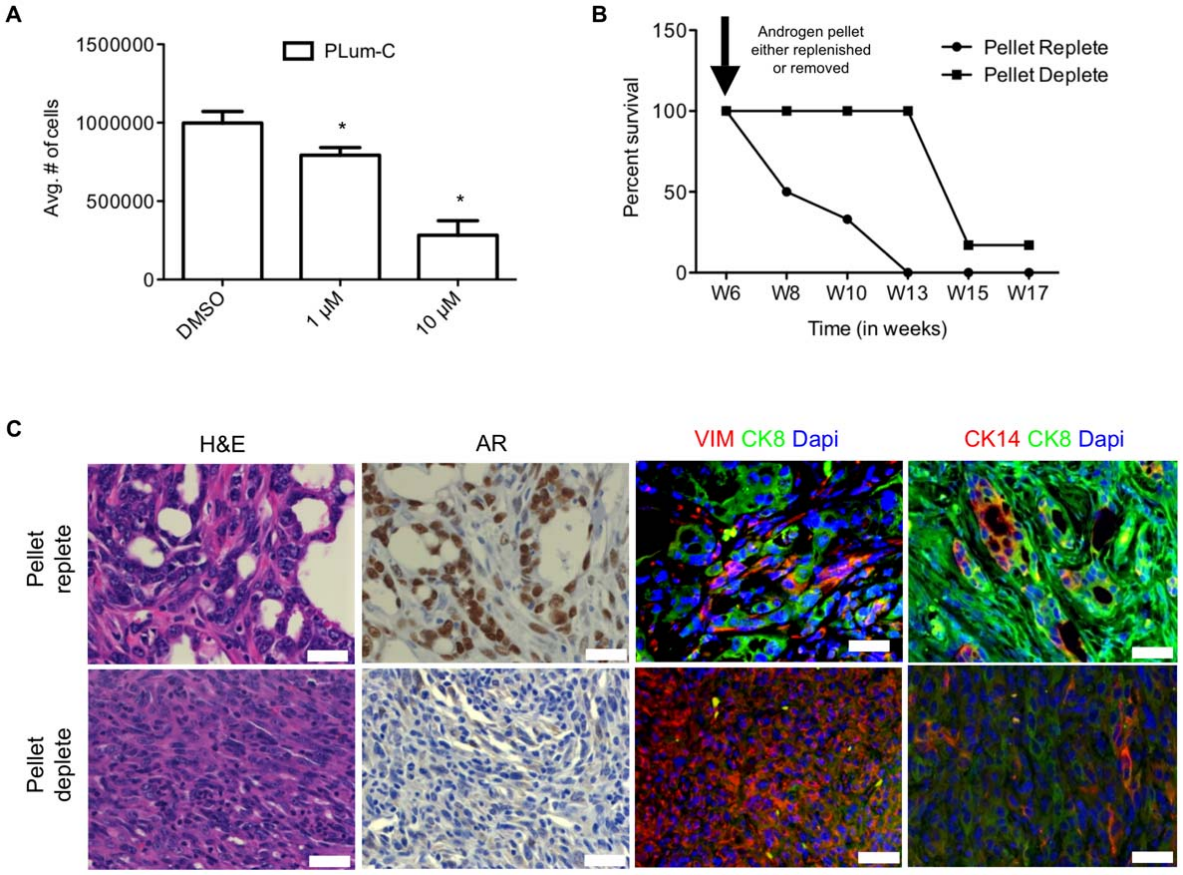
In vitro androgen dependence and in vivo castrate resistance of PLum-C cells. (**A**) Effect of Bicalutamide on the proliferation of PLum-C cells. PLum-C cells were incubated with the indicated concentrations of Bicalutamide or DMSO vehicle for six days in serum free PrEGM. The number of viable cells was determined by trypan blue exclusion. The data are reported as mean ± SD (*, *p*<0.05). (**B**) Orthotopic tumors were generated by injecting PLum-C cells in surgically castrated nude male mice with implanted testosterone pellet. At week six post injection, mice were randomly separated into two groups and testosterone pellets were either removed (androgen deplete) or replenished (androgen replete) in each group. Mice were sacrificed upon showing signs of morbidity and the survival rate was plotted. (**C**) Serial sections of orthotopic tumors were stained with H&E, AR, Vimentin, CK8, CK14, and Dapi. Scale bar  = 20 µm.

Because androgen-dependance is a typical characteristic of prostate adenocarcinoma, we sought to determine the behavior of cells under conditions of inhibited androgen receptor (AR) signaling. PLum-C cells (1×10^4^) were grown in serum-free prostate media in the presence of increasing concentrations of Bicalutamide, an AR antagonist, and 1 µM and 10 µM Bicalutamide resulted in 20% and 70% inhibition of growth, respectively (Fig. 6A). This suggests that AR function is required for sustaining in vitro cell proliferation in the majority of cells, even though exogenous androgen was not supplied. Subsequently, we investigated whether androgen ablation in vivo would delay morbidity from tumor burden as seen in clinical cases. Single cell suspensions of PLum-C cells were injected orthotopically into the prostates of castrated male nu/nu mice in the presence of an implanted testosterone pellet, and growth was monitored over time using bioluminescent imaging. Six weeks post injection, mice were randomly divided into two cohorts. Androgen pellets were removed from one group and replenished in the other group. The survival of mice with androgen supplementation ranged from 6 to 10 weeks. Animals switched to androgen deprivation conditions showed increased survival, ranging from 13 to 17 weeks (Fig. 6B). As shown previously in Figure 4A, androgen-supplemented orthotopic prostate tumors were comprised mainly of adenocarcinoma with minor components of solid/undifferentiated and sarcomatoid carcinoma (Fig. 4A, Fig. 6C and Table 1). This histological appearance was reversed in androgen-deprived orthotopic tumors, which were comprised of primarily sarcomatoid carcinoma (approximately 50–75% estimated tumor area), with the rest being solid/undifferentiated and less than approximately 10% adenocarcinoma (Fig. 6C). Regardless of the androgen status, all orthotopic tumors were highly invasive into the adjacent normal recipient prostate gland and stroma, and lymphovascular invasion was common. As in the adenocarcinoma, sarcomatoid carcinoma was also comprised of cells with a luminal (CK8^+^/CK14^-^/CK5^-^) or intermediate phenotype (CK8^+^/CK14^+^/CK5^-^), with the notable exception of the absence of CK8^+^/CK14^+^/CK5^+^ cells (Table 1). Sarcomatoid carcinomas demonstrated epithelial to mesenchymal transition as the cells displayed reduced CK8 expression (with occasional foci of CK8 loss) coupled with nearly ubiquitous vimentin co-expression. Synaptophysin expression was observed diffusely in a proportion of cells throughout all tumors regardless of histological pattern or androgen status. Thus, androgen deprivation selected for the growth of poorly differentiated cells co-expressing luminal, mesenchymal and neuroendocrine lineage markers. AR expression was strongly influenced by the androgen status of the recipient mouse. Orthotopic tumors in androgen-supplemented mice displayed strong nuclear labeling in adenocarcinoma with almost complete loss of nuclear labeling in sarcomatoid carcinoma. In both patterns there were occasional foci with loss of nuclear AR. Androgen deprivation led to almost complete loss of nuclear AR in all tumors, with only rare foci containing low cytoplasmic expression. As in the androgen-replete tumors, small undifferentiated lung metastases were observed in 3/6 androgen-deprived mice, with no notable differences in the differentiation markers when compared to lung metastases from androgen-replete and depleted metastases.

## Discussion

We describe here the in vitro origination from protospheres of a prostate epithelial cell line and clonal derivative that give rise to adenocarcinoma tumors and display properties of progressive prostate cancer, including metastatic potential and castrate-resistant growth. Here, we have directly established a cell line from progeny resulting from the initiation of *Pten/TP53* tumor suppressor loss in prostate epithelial stem/progenitor cells. In stem cells, TP53 controls the generation of self-renewing daughter stem cells and suppresses pluripotency [25]. PTEN negatively regulates cell cycle entry via AKT-dependent and AKT-independent pathways [26], [27], [28]. The combined loss of *Pten* and *TP53* results in robust prostate progenitor amplification, consistent with rapid and heterogeneous tumor development in the Pb-Cre4;*Pten^fl/fl^;TP53^fl/fl^* mouse model [24], [25].

The PLum-P/C lines grown in vitro have a CK8^+^/CK5^-^/NKX3.1^+^/AR^+^ luminal epithelial phenotype. Importantly, a population of cells has retained multipotency and/or differentiative plasticity. This is demonstrated in clonally-initiated protospheres by the presence of multiple cell types displaying various differentiation markers including intermediate cytokeratin phenotypes, CK8^+^/CK5^+^ and CK8^+^/CK14^+^, and by β3-tubulin/CK8^+^ cells. Similarly, we observed in PLum-P/C tumors, cell populations displaying intermediate cytokeratin profiles, neuroendocrine markers, and occasionally the definitive basal marker, TP63. These results parallel the expectations for multipotent self-renewing progenitor cells that mainly differentiate to luminal cells, suggesting that the PLum model will be useful to investigate the cancer stem cell model of prostate cancer. To date there is limited knowledge concerning the differentiation status and correlative tumorigenic and metastatic properties of prostate cancer tumor initiating cells [5], [16]. In one recently described model, cell fractionation studies of CWR22 human prostate cancer xenograft tumors demonstrated that tumor protospheres containing CK8^+^/CK5^+^/AR^-^/TP63^-^ cells gave rise to CK8^+^/CK5^-^/AR^+^ tumors [10]. Taken together, these data support the hypothesis that immature progenitor cells give rise to adenocarcinoma.

PLum-P/C tumors recapitulate many properties of human PC, i.e. clinical progressive adenocarcinoma. The capacity of PLum cells to metastasize from an orthotopic tumor or following intracardiac inoculation is relatively rare among prostate cancer cell lines. Interestingly, metastatic colonization appeared to initiate from progenitor cells with differentiation capability since both CK8^+^/CK14^+^ and CK8^+^/CK14^-^ cells were observed in individual metastases. The lack of AR in metastases also suggests a relatively immature metastasis-initiating cell. In addition, following intra-tibial inoculation, PLum-C cells formed tumors that elicited an osteoblastic response, characteristic of clinical prostate cancer metastasis [1], [2]. Bone is the most common site of prostate cancer metastasis, and therefore understanding the molecular mechanisms that promote prostate cancer metastasis to bone is a priority. Most existing prostate cancer intratibial models lead to osteolytic metastases, which has limited the ability to investigate the interaction of PC cells with the bone microenvironment to elicit osteoblastic responses [29].

PLum-C tumors regressed in response to androgen deprivation, consistent with an AR^+^ luminal phenotype for the majority of tumor cells. We observed subsequently arising castration-resistant tumors that had significantly decreased AR levels and had undergone an EMT to produce tumors containing many CK8^+^/Vimentin^+^ cells. It is not clear whether AR signaling has an active role in suppressing EMT, or alternatively, spontaneously-arising sarcomatoid cells may selectively survive androgen deprivation.

In conclusion, here we demonstrate the establishment of a cell line from a pre-enriched source of progenitor cells containing deletions of *Pten* and *TP53*, genetic aberrations associated with progressive human prostate cancer. These cell lines recapitulate the many aspects of the human disease, namely the formation of metastatic adenocarcinoma. While other prostate cancer cell lines have been established both from primary tumors and from metastases, to our knowledge this is the first prostate cancer cell line to utilize such an approach. This approach allows for a more detailed understanding of the potential contribution of progenitor cells to properties of progressive prostate cancer, and this model adds to the resources available to investigate the molecular mechanisms contributing to prostate tumor progression and metastasis. For example, models to test therapeutic approaches to treat osteoblastic bone metastasis are an area of current unmet need.

## Materials and Methods

### Ethics Statement

All animal experiments were done according to the protocol (LCB-018) approved by the National Cancer Institute Guideline for Use and Care of Animals.

### Cell Culture

#### Reagents

PrEGM with supplements was from Lonza (Lonza, MD), and DMEM containing 10% FBS and antibiotic reagents were purchased from Invitrogen (Invitrogen, CA). Dispase, collagenase type II and trypsin were purchased from Invitrogen. BD Matrigel™ was purchased from BD Biosciences (BD Biosciences, CA). Bicalutamide was from Sigma-Aldrich (Saint Louis, MO).

#### Preparation of Primary Mouse Prostate Epithelial Cells

Three to five month old *Rosa26-Cre^ER^Pten^fl/fl^P53^fl/fl^* mice were euthanized by carbon dioxide inhalation and the entire lower urogenital tract aseptically removed and placed in 35-mm Petri dishes in prostate harvest media (DMEM containing 10% FBS, 1% penicillin/streptomycin, gentamicin (300 µg/ml), and amphotericin B (0.5 µg/ml). All prostate lobes were micro-dissected away from the remaining urogenital tract tissues, pooled and placed in 35-mm Petri dishes in prostate dissection media (prostate harvest media with collagenase type II at 1 mg/ml). Prostatic tissue was finely minced using a sterile scalpel blade, collected, and incubated in prostate dissection media on a spinning wheel for 1 hr at 37°C. Tissue suspensions were centrifuged for 6 min at 1000 rpm, and the pellet (organoids) was resuspended in at least 0.5 ml of 0.05% trypsin-EDTA and incubated for 5 min at 37°C. The organoid/trypsin suspension was carefully passed through 19, 23, 25, 27.5, and 31 gauge needles before the trypsin was neutralized using prostate harvest media. To confirm that a single cell suspension was achieved, an aliquot of the cell suspension was placed on a glass slide and the integrity and separation of cells was examined under a microscope. The cell suspension was centrifuged for 6 min at 1000 rpm and washed once with serum free prostate epithelial cell basal media, PrEGM (supplemented as directed by the manufacturer with bovine pituitary extract, insulin, hydrocortisone, gentamicin, amphotericin B, retinoic acid, transferrin, T3, epinephrine, recombinant human epidermal growth factor, and penicillin/streptomycin). After washing, cells were passed through a 40 µm cell strainer and centrifuged for 6 min at 1000 rpm. Finally, the pellet was resuspended in 1 ml of PrEGM and the number of viable cells was counted using the trypan blue exclusion method.

### Establishment of de novo *Pten^-/-^/TP53^-/-^* mouse prostate epithelial cell lines

#### 3-D Cultures and Sphere-formation Assay

Single cells were suspended in Matrigel™/serum free PrEGM (1∶1) at a concentration of 10,000 cells/well in a total volume of 100 µl, in triplicate. The solution was plated gently around the rim of individual wells of a 12-well plate and allowed to solidify for 1 hr at 37°C in a humidified incubator. Tamoxifen-containing serum free PrEGM was added gently to the center of each well and the media was changed every two to three days. Spheres were harvested between days 12 and 15 after plating.

#### Propagation of spheres

Media was gently aspirated from the center of the well, and the Matrigel™ was digested by incubation in 500 µl of serum free PrEGM containing dispase (1 mg/ml) for 1 hr at 37°C in a humidified incubator. The resulting sphere containing solution was collected and centrifuged at 1000 rpm for 6 min. The resulting pellet was resuspended in 500 µl of serum free PrEGM containing collagenase II (1 mg/ml) and incubated for 1 hr on a spinning wheel at 37°C. The suspension was centrifuged at 1000 rpm for 6 min and was resuspended in 500 µl of 0.05% trypsin-EDTA. The solution was directly passed through a series of needles (21, 25, 27 and 31 gauge). Trypsin was inactivated by adding PrEGM media containing 5% FBS, and cells were centrifuged at 1000 rpm for 6 min, washed once and resuspended in serum free PrEGM. The number of viable cells was determined using the trypan blue exclusion method. Cells were then resuspended in Matrigel™ and plated as described before (10,000 cells/well). Spheres were propagated for three generations and enriched progenitor cells were then switched to 2-D cultures.

#### 2-D Cultures

Single cell suspensions obtained from Generation 3 spheres as described above were seeded in 6-well plates in PrEGM containing 2.5% FBS at a density of 20,000 cells/well in triplicate and incubated at 37°C in a humidified incubator. After a monolayer of epithelial cells was formed, cells were trypsinized, counted and plated as before. After growing in PrEGM containing 2.5% FBS for 5 passages, the serum concentration was gradually decreased to 0.5 and then to 0. Cells with CK5^+^/CK8^+^ expression were detected in early passages and while in serum. No CK5 expression was detected once cells were transferred to serum-free media. All the experiments shown were carried out with cells grown in serum free PrEGM. Plum-P (Prostate Luminal-Parental) refers to the parental cell line and Plum-C (Prostate Luminal-Clone) refers to a clonal daughter cell line generated from Plum-P cells.

### Immunofluorescence and Confocal Microscopy

#### Antibodies and Reagents

Antibodies from the indicated manufacturers used in this study were as follows: mouse monoclonal anti-CK8, rabbit polyclonal anti-CK14, and rabbit polyclonal anti-CK5 (Covance, CA), rabbit polyclonal anti-β3 tubulin (Millipore, CA), rabbit polyclonal anti-synaptophysin and rabbit polyclonal anti-NKX3.1 (Abcam, MA), rabbit polyclonal anti-AR (Santa Cruz Biotechnology, CA), Alexa 488 goat anti-mouse, goat anti-rabbit, Alexa 568 goat anti-mouse, goat anti-rabbit (Invitrogen, CA). Fluoro-gel II with Dadi was purchased from EMS (Electron Microscopy Sciences, PA).

#### Staining Procedures for monolayer cells

Adherent cells were fixed in 4% paraformaldehyde (PFA) in PBS for 10 min, followed by permeabilization with 0.5% Triton X-100 in PBS for 2 min. Non-specific sites were blocked by incubation in 2% BSA in PBS for 30 min. Cells were then incubated overnight at 4°C with the specified antibodies in 2% BSA/PBS. Cells were washed three times with PBS containing 0.1% Tween-20, incubated with Alexa-488 and/or 568 conjugated IgG in 2% BSA for 30 min at room temperature, and finally washed and mounted using the anti-fade reagent Fluoro-gel II with DAPI. Fluorescent signals and bright field images were captured using an inverted upright fluorescent Zeiss Axioplan microscope and Zeiss LSM 510 Meta Mk4 confocal microscope.

#### Staining Procedures for Protospheres

Cells were grown in 35 mm glass bottom culture plates with 10 mm microwell (MatTek Cultureware, MA) in Matrigel™-containing media as described above. Spheres were fixed in-situ in 4% PFA at room temperature for 20 min. The PFA was aspirated gently and spheres were permeabilized with 0.5% Triton X-100 for 30 min at room temperature. After carefully aspirating the permeabilization solution, spheres were blocked using the sphere blocking buffer (0.1% BSA, 0.2% Triton X-100, 0.05% Tween-20, and 10% normal goat serum in PBS) for 2 hrs at room temperature. Spheres were incubated overnight with primary antibodies at 4°C. After gentle washing with PBS containing 0.1% Tween-20, spheres were incubated with Alexa-488 and/or 568 conjugated IgG for 2 hrs at room temperature. The culture plates were then washed gently, and 50 µl of the anti-fade reagent Fluoro-gel II with DAPI was added directly on the microwell and a 12-mm glass coverslip was gently mounted. Confocal microscopic analyses were performed using Zeiss LSM 510 Meta Mk4 confocal microscope and images were acquired and analysed using the Zeiss LSM image software.

### Histology and Immunohistochemistry

Subcutaneous tumors and brain and lung tissues were harvested and fixed in 4% PFA overnight, rinsed well in PBS, and transferred to 70% ethanol before standard processing to obtain paraffin-embedded sections. Unstained tissue sections were deparaffinized, and antigen retrieval was performed in a citrate buffer (DAKO targeted antigen retrieval solution) in a steamer at 100°C for 15 min followed by 15 min incubation at room temperature. Blocking was performed with Cyto Q Background Buster reagent (Innovex Biosciences, Richmond, CA) for 30 min at room temperature. Primary antibody incubation was performed overnight at 4°C, followed by secondary antibody incubation at room temperature for 30 min. The ABC peroxidase kit (Vector Laboratories, Burlingame, CA) was used followed by DAB (Dako, Carpinteria, CA) for chromogen visualization. All slides were counterstained with hematoxylin.

### In Vivo Transplantation

#### Subcutaneous Transplantation

A total of 1×10^6^ PLum-P or PLum-C cells in 50 µl was mixed 1∶1 with growth factor reduced Matrigel™ (Becton Dickinson) immediately prior to injection. Cells were injected subcutaneously into the flanks of 8–10 week old BALB/c *nu/nu* male mice. Mice were euthanized upon signs of morbidity.

#### Orthotopic Transplantation

8–10 week old BALB/c *nu/nu* male mice were anesthetized and a midline skin incision was made. Mice were surgically castrated prior to cell inoculation. PLum-C cell suspensions (500,000 cells in 10 µl prostate harvest media) were injected using a 30-gauge needle, a 50 µl Hamilton syringe, and a surgical micromanipulator with a microsyringe pump (World Precision Instruments) into one of the anterior prostatic lobes by gently retracting the bladder and seminal vesicles caudally with a sterile moist cotton swab. Standard surgical procedures were used to repair the abdominal musculature and incision. Testosterone pellets (5 mg) were surgically implanted subcutaneously intrascapular at the time of castration. All procedures of the operation described above were performed under a bio-hood with a surgical microscope (World Precision Instruments). Bioluminescent imaging was used biweekly to monitor the growth of tumors and mice were euthanized when they showed signs of morbidity.

#### Intracardiac Transplantation (Experimental Metastasis)

A total of 1×10^5^ PLum-P or PLum-C cells in 100 µl of PBS were inoculated into the left cardiac ventricle of male nude mice under anesthesia. Bioluminescent imaging was determined bi-weekly to monitor the development of metastasis. Mice were euthanized after demonstrating signs of morbidity and tissues were collected and fixed in 4% PFA for histological analysis (HistoServ, Inc.).

#### Intratibial Transplantation

Mice were anesthetized, the knees were flexed to a 90° angle, and a 27-gauge needle was inserted through the tibial plateau. A Hamilton syringe was used to accurately inject 25 µl of PLum-C cells in PBS (1×10^5^) into the proximal tibia. Radiography was determined bi-weekly using an MX-20 Faxitron x-ray system (Faxitron) to monitor the growth of tumor and sign of lesions in bone. Mice were euthanized when bone lesions were detected upon X-ray examination. Tibial bones were collected and fixed in 4% PFA for histological analysis.

### In Vitro Proliferation Assay

PLum-C cells were seeded in triplicate in 6-well plates at a density of 1×10^4^ cells per well. Cells were grown for six days in serum-free PrEGM in the presence of DMSO or DHT (5 nM) or Bicalutamide (1 µM and 10 µM). Cells were then trypsinized and counted using trypan blue exclusion method.

### Reverse Transcription-PCR

Total RNA was isolated from cells using the RNeasy Micro Kit (Qiagen) according to the manufacturer's instructions. cDNA was generated from total RNA using the Super Script III First Strand Synthesis System for RT-PCR (Invitrogen). PCR was performed using Platinum Taq Polymerase (Invitrogen). SYBR Green Mastermix was used for quantitative RT-PCR on the Stepone Plus RT-PCR system (Applied Biosystems). All reactions were run in triplicate using the primers listed below, designed on Genescript or Primer 3. All values were normalized to Actin. The primers used were: Actin-F: 5′TCCTCCCTGGAGAAGAGCTA3′; Actin-R: 5′ACGGATGTCAACGTCACACT3′. *Pten*-F:5′TTGAAGACCATAACCCACCA3′; R:5′TTACACCAGTCCGTCCCTTT3′. *TP53*-F:5′TGGTGGTACCTTATGAGCCA3′; R: 5′AGGTTCCCACTGGAGTCTTC3′.

### Data analysis

The significance of the data was analyzed using a Student's *t*-test, and differences between two means with a *P*<0.05 were considered significant. Error bars represent the standard error of the mean (SEM).

## Supporting Information

Figure S1 ***Pten^-/-^TP53^-/-^*** **cell lines form adenocarcinoma in vivo and express various differentiation markers.** (A) Subcutaneous tumor was generated by injecting male nu/nu mice with PLum-P and PLum-C cells. Cross sections of subcutaneous tumors stained with H&E showing typical adenocarcinoma generated by PLum-P (left panel) and PLum-C (right panel) cells are shown. Scale bar  = 10 µm. (B) representative sections of PLum-C subcutaneous tumor stained for CK5, CK8, CK14, AR, TP63, Synaptophysin, and Dapi. Scale bar  = 10 µm.
(TIF)

Figure S2 **The tumorigenic origin of Synaptophysin+ cells in Plum-C orthotopic tumors.** A representative section of Plum-C orthotopic tumors co-stained for Synaptophysin, P-AKT, and Dapi. Scale bar  = 10 µm.
(TIF)
