# Supplementary figures and images for: Self-Renewing Pten-/-TP53-/- Protospheres Produce Metastatic Adenocarcinoma Cell Lines with Multipotent Progenitor Activity
Source: PLoS One. 2011 Oct 11;6(10):e26112. doi: 10.1371/journal.pone.0026112 (PMC3191168; doi:10.1371/journal.pone.0026112)

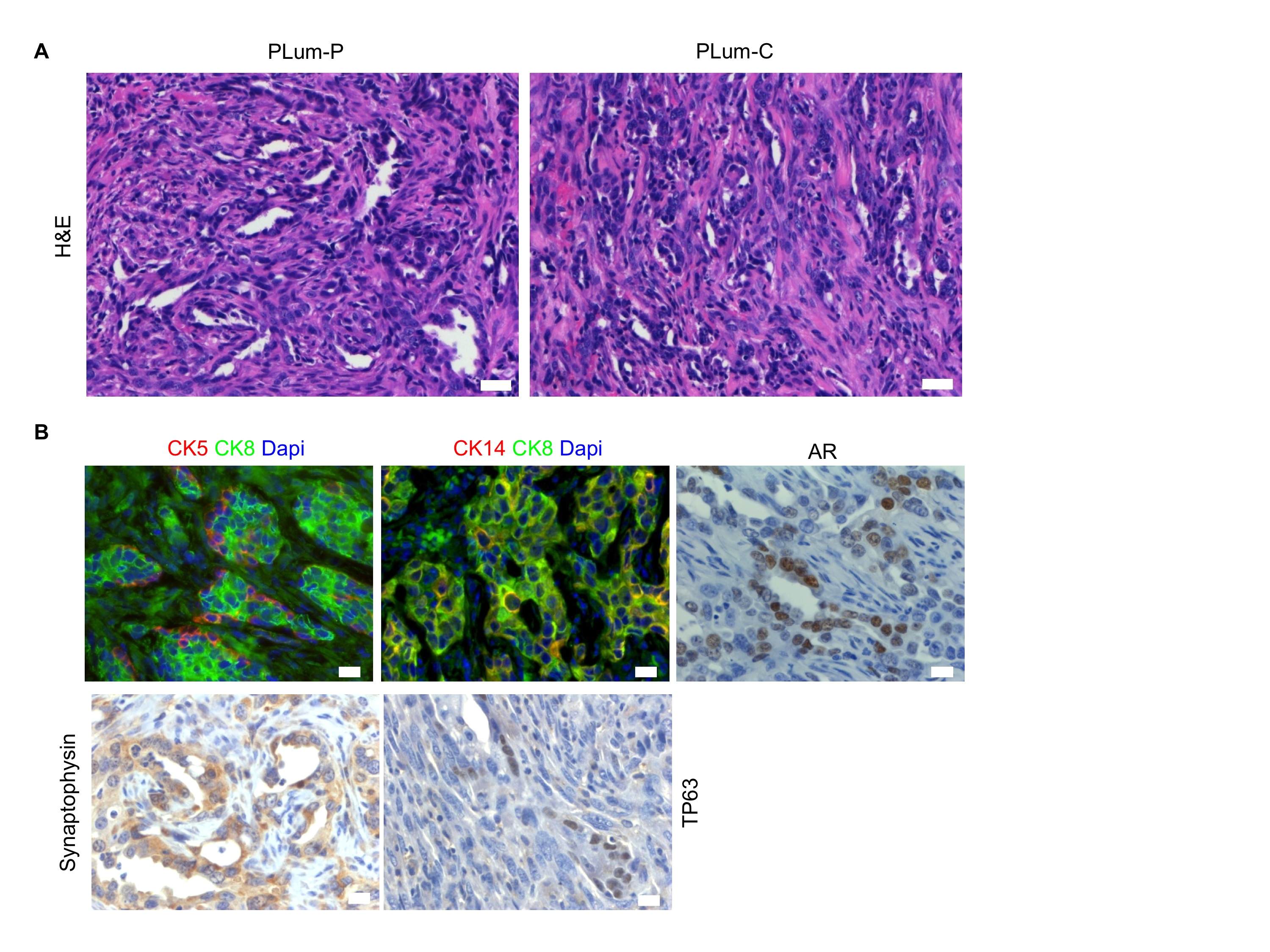

Supplement: Figure S1 — Pten-/-TP53-/- cell lines form adenocarcinoma in vivo and express various differentiation markers. (A) Subcutaneous tumor was generated by injecting male nu/nu mice with PLum-P and PLum-C cells. Cross sections of subcutaneous tumors stained with H&E showing typical adenocarcinoma generated by PLum-P (left panel) and PLum-C (right panel) cells are shown. Scale bar = 10 µm. (B) representative sections of PLum-C subcutaneous tumor stained for CK5, CK8, CK14, AR, TP63, Synaptophysin, and Dapi. Scale bar = 10 µm. (TIF) [file pone.0026112.s001.tif]

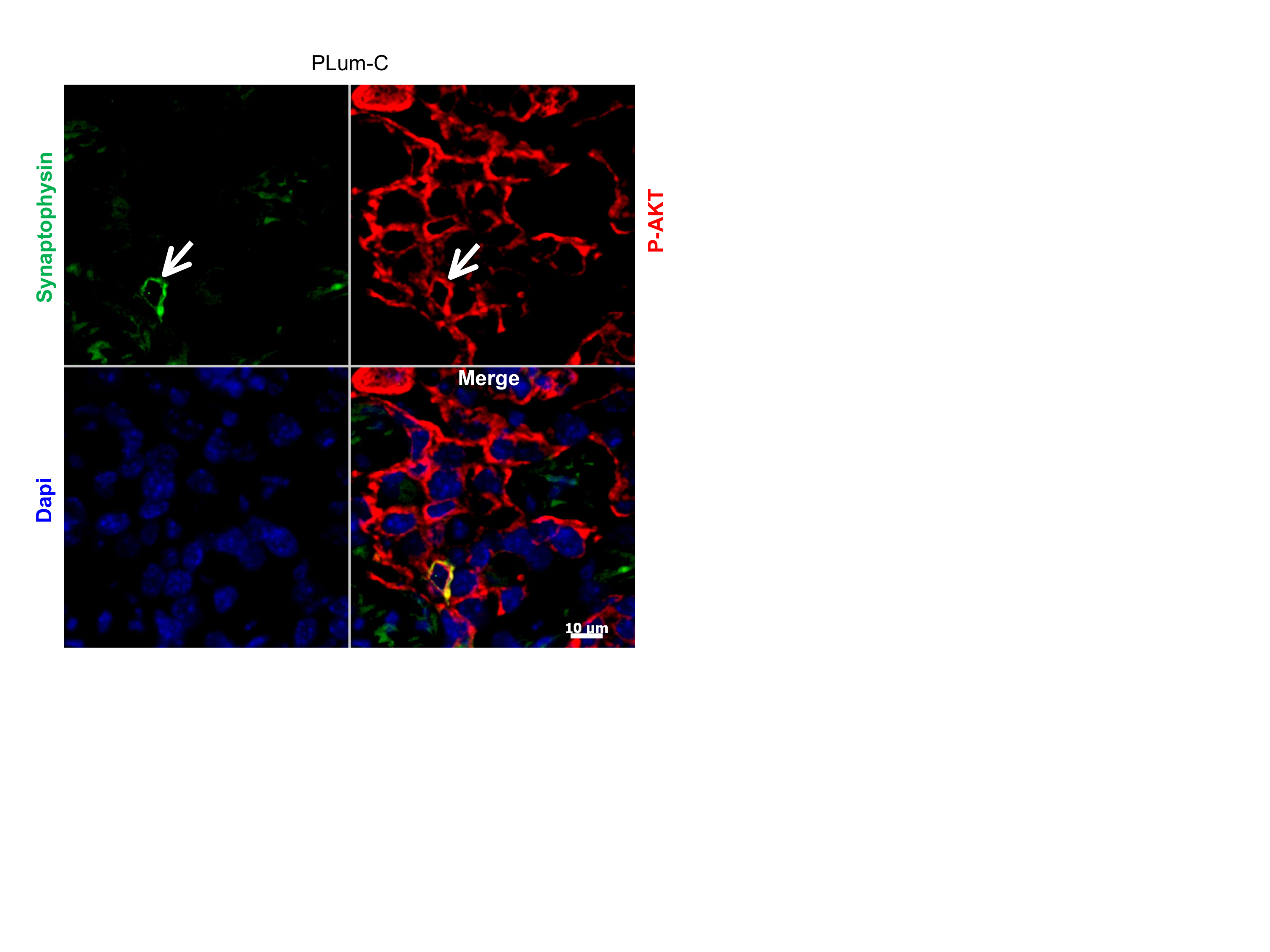

Supplement: Figure S2 — The tumorigenic origin of Synaptophysin+ cells in Plum-C orthotopic tumors. A representative section of Plum-C orthotopic tumors co-stained for Synaptophysin, P-AKT, and Dapi. Scale bar = 10 µm. (TIF) [file pone.0026112.s002.tif]
